# Elder Abuse—The Current State of Research in South Africa

**DOI:** 10.3389/fpubh.2018.00358

**Published:** 2018-12-04

**Authors:** Carla Kotzé

**Affiliations:** Department of Psychiatry, Faculty of Health Science, School of Medicine, Weskoppies Psychiatric Hospital, University of Pretoria, Pretoria, South Africa

**Keywords:** elder, abuse, neglect, South Africa, marginalization

## Abstract

**Objectives:** The concern about elder abuse increases as the global population ages. Elder abuse is a global public health, human rights, and criminal justice problem that goes beyond socioeconomic classes and regions. It remains understudied, especially in developing countries with limited resources. It is therefore timely to do a review of the available research on elder abuse in South Africa, to be able to address the gaps in the research with future projects.

**Methods:** Publications were identified from PubMed, MEDLINE, PsychINFO, Sabinet, Africa-Wide Information, CINAHL, E-Journals, Family and Society Studies Worldwide, PsycARTICLES, Criminal Justice Abstracts, and Social Work Abstracts. Fourteen articles on elder abuse in South Africa were selected for further review. This paper provides a narrative review of elder abuse in South Africa and is not a systematic review.

**Discussion:** South Africa is a multicultural nation, plagued by HIV/AIDS, poverty, and the remaining effects of the legacy of apartheid. This background sets the stage for categories of abuse that are unique to the country that are highlighted in this review. The available research on elder abuse is very limited and no reliable data about the prevalence of elder abuse in South Africa could be found.

**Conclusions:** There is a clear need for more longitudinal research about all aspects of elder abuse in South Africa. To improve future research efforts consensus has to be reached about a universal operational definition of abuse and an elder abuse instrument with a clear cut-off for definite elder abuse.

## Introduction

As the global population ages, the number of people aged 60 years and older is estimated to reach 2 billion by 2050. This increase in the older population heightens the concern about elder abuse and neglect. Elder abuse is a global public health, human rights, and criminal justice problem. It goes beyond, socioeconomic classes, regions, languages, and ethnicities, but remains understudied, especially in developing countries with limited resources. Available evidence suggests that elder abuse is prevalent, predictable, costly, and sometimes fatal (1).

Published figures of elder abuse and neglect varies greatly with ranges between 2 and 60%. An estimated overall prevalence of elder abuse of ~10% is considered reasonable, but is likely to be an underestimate. The prevalence of abuse in institutional settings are considered to be higher, with up to 64.2% of staff admitting to elder abuse based on self-report. Research has suffered from several weaknesses and has been hindered by the lack of consensus on a single universal operational definition of elder abuse and neglect, measurement problems, and inconsistent research methodologies (1–4).

The definition offered by Action of Elder Abuse in the United Kingdom and World Health Organization is: “a single or repeated act, or lack of appropriate action, occurring within any relationship where there is an expectation of trust which causes harm or distress to an older person.” Initial definitions were considered overly broad, however consensus has arisen about the inclusion of five major types of elder abuse: physical abuse, psychological or verbal abuse, sexual abuse, financial exploitation, and neglect (2, 3).

South Africa has a multicultural population with 4.2 million older persons and one of the most rapidly growing aging populations in Africa. Poverty remains widespread in the country and is exacerbated by high unemployment rates and effects of the HIV/AIDS epidemic. These factors create a scenario where specific types of abuse occur that are not covered by definitions used in developed countries. The single definition of what exactly constitutes elder abuse, with a clear cut-off for definite abuse has been equally elusive in South Africa (5, 6).

A systematic review done in 2015 indicated that elder abuse research should include developing countries and culturally diverse populations (1). It is therefore considered timely to provide and overview of the available research on elder abuse in South Africa, to be able to address the gaps in the research with future projects.

## Methods

To determine the status of elder abuse research in South Africa, a narrative review was done. An expert reference librarian searched the global literature in PubMed, MEDLINE, PsychINFO, Sabinet, Africa-Wide Information, Cumulative index to Nursing and Allied Health Literature (CINAHL), E-Journals, Family and Society Studies Worldwide, PsycARTICLES, Criminal Justice Abstracts, and Social Work Abstracts. The databases were searched using combinations of the following keywords: abuse, aged, elderly, elder abuse, assault, exploitation, maltreatment, neglect, South Africa.

The inclusion criteria were: English-language articles reporting on abuse of people aged 60 years and older in South Africa. A ministerial committee of inquiry was set up to investigate alleged elder abuse during 2000–2001. After the two volumes report of the findings was published, awareness of elder abuse increased leading to changes in policy and legislation in South Africa. For this reason only articles published since 2000 were included (7).

The researcher reviewed each of the 39 citations and abstracts that were retrieved. After this the full texts were retrieved and reviewed. A manual search of the reference list of all the included articles was done. All peer-reviewed articles/theses that were deemed to be elder abuse research or reviews in a South African population were included. Newspaper articles, case studies, government reports, textbook chapters, conference papers, and other publications that were not peer-reviewed were omitted.

## Results

**Figure 1:**
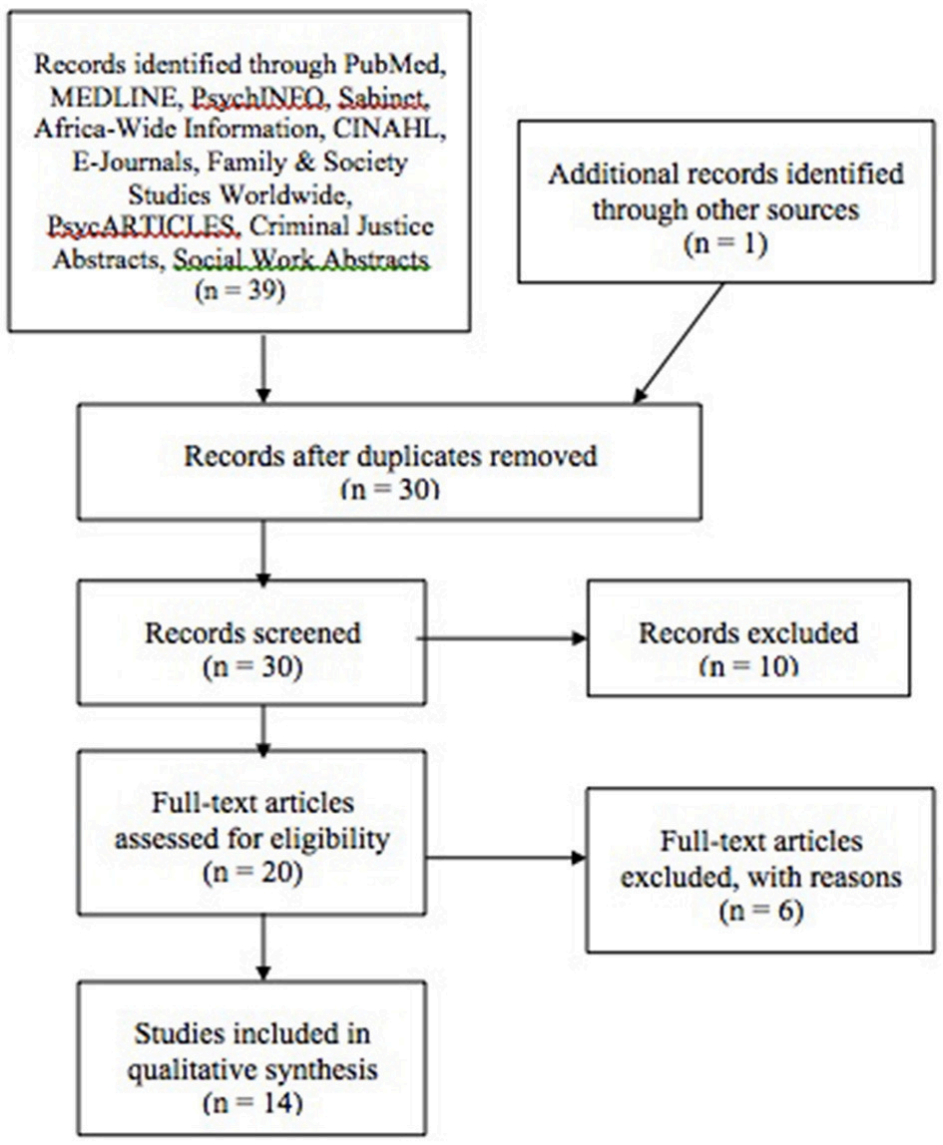
Flow diagram of review inclusions and exclusions. Source:Moher et al. (8).

The initial search results resulted in a total of 39 papers; one additional study was added after manual search of the reference lists of the publications that were already included. Refer to Figure 1 for a flow diagram of the search results (8).

Two studies were excluded because they were done in Namibia and one study was published before 2000. Screening of the titles and abstracts excluded seven more studies, as they were found irrelevant to the review scope. The full texts of the remaining 20 articles were retrieved for final assessment. After applying the inclusion and exclusion criteria six more articles were excluded, because they were not original research or reviews. Fourteen articles were included.

Ten articles were published in peer-reviewed journals and contained original data or reviews on elder abuse in South Africa. The publications of four research projects, where a thesis was written for master degree purposes, were also included. Most of the studies included used qualitative or review methods.

There were only four studies with original research of a quantitative nature: the study by Bigala and Ayiga included cross-sectional data of 506 elderly persons (9); Meel did a retrospective descriptive study that included 2,579 adult females of which 145 (5.6%) were elderly (10); Makiwane and Kwizera used a representative sample of 900 randomly selected elderly persons and did structured interviews and direct observations (11); and Marais et al. combined qualitative methods with analysis of data collected by a telephone-based service to report abuse and included data from 3,402 calls over a 2-year period (6).

## Discussion

There have been several systematic reviews about the state of elder abuse globally. The majority of studies of elder abuse were conducted in North America, Europe, and Asia, with only two studies identified in Africa. Five of the most recent reviews of elder abuse research, done between 2011 and 2018, and one review that focused specifically on sexual violence against older people did not include any studies done in South Africa (1–4, 12, 13).

The proportion of elderly in South Africa is on the increase with 8.1% of the population being over the age of 60-years. Despite this, the problem of elder abuse has not received much attention from researchers or policy makers. No national epidemiological studies about elder abuse in South Africa could be found (14).

Only one study included recent data about the prevalence rates of elder abuse in a small local area and another study focused specifically on rates of sexual assault in elderly woman. From these studies it appears as if the rates of abuse are very high. Bigala and Ayiga found that 64.3% of men and 60.3% of women reported experiences of abuse in Mafikeng. Mafikeng is situated in the Northwest province of South Africa with an estimated population of 250,000. In this study it was found that men experienced more physical abuse and women more emotional, financial, and sexual abuse. In Mthatha, between 2007 and 2011, the average rate of rape among elderly women was 20.7/10 000 adult women per year (9, 10).

Preliminary analysis of 3,402 calls over a 2-year period to a national toll-free service to report abuse showed that the biggest category of reported abuse was physical abuse, followed by financial abuse and problems with pensions. Two-thirds of the reported incidents took place in private homes and in half of the cases the victim was the caller (6).

As found in the international literature, local research into elder abuse is hindered by the lack of a universal operational definition with a clear cut-off for what exactly constitutes abuse. The definitions used by organizations like Action on Elder Abuse South Africa, National Strategy on Elder abuse and Department of Social Development all vary slightly. It is also different from the definition as provided in the Older Persons Act 13, of 2006 where it is defined as “any conduct or lack of appropriate action, occurring within any relationship where there is an expectation of trust, which causes harm or distress or is likely to cause harm and distress to an older person.” It specifies the types of abuse as physical, sexual, psychological, and economic (15–17).

Against the background of the historical disadvantages and the multicultural nature of the South African population, this definition is sometimes considered too narrow. There exist unique categories of abuse not usually found in developed countries. These include loss of respect for elders and systemic abuse, which refers to dehumanizing treatment at health clinics, pension pay points, and marginalization of older people by the government. There are also problems with allegations of witchcraft, mainly against elderly black women, and as a result of the accusations their property can be confiscated and they can be assaulted or even burnt. Another concerning tendency is particular violent forms of abuse, specifically rape of older women by sons or grandsons to extort pension money. These acts tend to be concealed and are often not seen as rape in the communities, because the abuse is perpetrated within the family (5, 17, 18).

In addition to these challenges, South Africa had to deal with the effects of the HIV/AIDS epidemic as the country with the highest number of infected people in the world. During 2017 it was estimated that 7.06 million South Africans or 12.6% of the total population were HIV positive, with young, black females being most at risk. HIV/AIDS has not only changed the roles of older persons to being caregivers and providers for ill adult children or orphaned grandchildren, but has also traumatized them. They suffer from the effects of caregiving, the death of relatives who succumb to AIDS and the grief and isolation attached to the illness. Secondary to the illness or death of family members, entire households end up being dependent on the pensions of older people (11, 19, 20).

The Older Persons Act came into effect in April 2010 and aims to maintain and protect the constitutional rights of all older persons and to facilitate accessible, equitable, and affordable services to older persons. The Act makes provision for mandatory reporting of suspected abuse by any citizen, not just by health care professionals, but the procedures for reporting remains cumbersome. The Department of Social Development also had to establish a register to keep track of criminals who have committed crimes against elder persons. This register is still not up and running. There is a lack of enforcement mechanisms for the various sets of rights conferred on older persons by the Act and law enforcement agencies often fail older victims who attempt to report abuse (18, 21–23).

The majority of older South Africans are not in residential care and there is a critical shortage of proper facilities to care for the elderly. Most of the elderly live in rural areas and in communities with their families or on their own. The care of older persons is regarded as the responsibility of the family and the state has made it clear that its duty to provide care applies only in the case of frail older persons who have no family to care for them. The expectation that elderly should be cared for by the younger generations has in some cases been turned upside down because of rapid social change, urbanization, migration, and the high incidence of HIV/AIDS (19, 22).

The main risk factors to become a victim of abuse are being female, presence of cognitive impairment, and being older than 74-years old. In the Mafikeng study factors that were associated with elder abuse included having no working children, being single or living in an elderly couple family, living in rural areas, having a poor self-perception of health and having a disability (4, 9).

Unemployment rates in South Africa are estimated at around 26.7%. Older people view poverty, unemployment and the subsequent use of alcohol and drugs as contributing factors to abuse. The breakdown of family structures, loss of respect for elders, beliefs in witchcraft, high crime rates, including domestic violence and socio-economic inequalities remaining after the apartheid system are also blamed (11, 19, 21, 22, 24).

The experiences of the elderly and whether or not they experience abuse and neglect is largely influenced by their socio-economic circumstances. Old age is easier for some groups in the South African population, but more research is needed into the prevalence and risk factors for abuse in specific groups. Culture, political, economic, and social realities, together with negative societal attitudes toward older persons are exacerbating the vulnerability of the elderly to poverty, abuse, and violation of their human rights (5, 25).

### Limitations

The extent of research done in the field of elder abuse in South Africa is very limited and the original research was done in small local areas in the country. The number of individuals included was small, with the focus on predominantly one racial, cultural, or socioeconomic group. This limits the generalizability of the research findings, even on a national level. No consistent definition of abuse or an elder abuse instrument that clearly defines a cut-off for definite elder abuse was used. Most of the reviews that were referred to included policy documents and other types of documentation that were not peer reviewed or were not original research.

### Clinical Implications

According to the articles that were reviewed with prevalence estimates, elder abuse seems to be very common and widespread in South Africa. The most vulnerable groups seem to be those living in rural areas of a lower socio-economic class and they tend to be older and frail. Health and health-allied professionals should consider routine screening for abuse in this population when the older person has contact with health services.

The majority of the elderly are being cared for in communities and some very frail older persons are receiving care from untrained individuals, increasing their risk of becoming victims of abuse. This situation is exacerbated by the lack of trained health and social welfare personnel to supervise geriatric care and community welfare organizations (24). Training programs should be developed and implemented for all personnel and community members dealing with the elderly population. Interventions and treatment should be culturally sensitive, patient-centered, and evidence-based.

There are many barriers to the mandatory reporting requirements. Health professionals may be reluctant to report elder abuse and this reluctance should be addressed by streamlining of the reporting process and implementation of effective enforcement mechanisms after such reports are lodged. There is a legal obligation to report suspected abuse, but for health professionals the overriding concern must always be the safety of the patient and prevention of unnecessary suffering.

### Recommendations for Future Research/Approaches

It is crucial that nations take a multifaceted approach to ensure protection of older adults. For this to be done effectively, national longitudinal research about the prevalence, specific risk factors for elder abuse in different contexts and its consequences is needed. Research can be improved and made more generalizable by the use of a single universal operational and comprehensive definition of what constitutes abuse and neglect. Culturally sensitive tools to define and measure elder abuse, such as the screening tool developed by the South African Department of Health, Elder Abuse Screening Tool (EAST) should be validated and considered for use in future research.

Older people should be informed about their rights and how to access support services. Community members and health professionals should be aware of the reporting requirements and processes to follow. Awareness campaigns can be done in the forms of community workshops and media campaigns, while government involvement should be encouraged. Research into elder abuse should be encouraged and results should be used to address gaps in policies, legal processes, and service delivery.

## Conclusion

Despite some unique categories and differences, elder abuse in South Africa shares similarities with all other countries. The elusive standardized operational definition and clear cut-off for elder abuse is a global problem and limits generalization and application of research findings. Collaborations between researchers, communities, governments, and different countries can improve future research efforts.

Elder abuse occurs in every country and governments have a very important role to play in the care and protection of older citizens and should implement strategies that allow persons to age with security and dignity. This should include early recognition of elder abuse, effective reporting processes, adequately trained professionals and suitable care facilities. The problem of elder abuse cannot be properly solved if essential needs are not met. Opportunities should be created for older persons to participate in their societies and to alleviate poverty and its associated negative consequences of ill health and abuse. There should be coordinated efforts at the national level to preserve and protect the human rights of vulnerable aging populations and to prevent elder abuse in all its forms.

## Author Contributions

The author confirms being the sole contributor of this work and has approved it for publication.

### Conflict of Interest Statement

The author declares that the research was conducted in the absence of any commercial or financial relationships that could be construed as a potential conflict of interest.
